# Intussusception associated with pneumatosis cystoides intestinalis in a male adolescent: A case report

**DOI:** 10.1002/deo2.256

**Published:** 2023-06-15

**Authors:** Yugo Horiuchi, Kazuya Miyaguchi, Hisashi Matsumoto, Hideki Ohgo, Yoshikazu Tsuzuki, Hidetomo Nakamoto, Hiroyuki Imaeda

**Affiliations:** ^1^ Department of General Internal Medicine Saitama Medical University Saitama Japan; ^2^ Department of Gastroenterology Saitama Medical University Saitama Japan

**Keywords:** colonoscopy, hyperbaric enema, intussusception, oxygen therapy, pneumatosis cystoides intestinalis

## Abstract

Pneumatosis cystoides intestinalis is an uncommon disease that rarely causes intussusception. We report the case of a 16‐year‐old male patient with intermittent abdominal pain who was diagnosed with intussusception. The patient had no history of raw food ingestion, fever, diarrhea, or hematochezia. Computed tomography revealed intussusception characterized by a crab‐finger appearance, and pneumatosis cystoides intestinalis was diagnosed by colonoscopy. Treatment with hyperbaric enema and low‐flow oxygen therapy resulted in a prominent improvement of the lesion. No recurrence was observed for > 1 year. Intermittent abdominal pain without diarrhea or hematochezia in male adolescents may represent pneumatosis cystoid‐related intussusception, and the addition of low‐flow oxygen therapy may help avoid surgery.

## INTRODUCTION

Pneumatosis cystoides intestinalis (PCI) is a rare disease, with a reported incidence of 0.03%. PCI is characterized by multiple emphysematous cysts forming in the submucosa and serosa of the intestinal wall.^1^

Its etiology remains unknown. However, several theories have been proposed, including the mechanical theory, which suggests that gas invades the intestinal wall via damaged mucosa^2^; the bacterial theory, which implicates gas‐producing organisms^3^; and the pulmonary disease theory, which suggests that gas originating from mediastinal emphysema, caused by ruptured alveoli in chronic lung disease, is released into the intestinal tract along the aorta and the mesenteric vessels.^4^ The clinical presentation of PCI includes abdominal pain, hematochezia, and diarrhea. Recent advances in imaging technology have led to new diagnostic techniques, increasing the number of incidental cases. Here, we describe a rare case of intussusception caused by PCI.

## CASE REPORT

A healthy 16‐year‐old male adolescent presented with intermittent stabbing abdominal pain that began the morning before admission to the emergency room. The pain did not improve despite using an over‐the‐counter H2 receptor antagonist. The pain worsened that night, and the patient was referred to our hospital the next day after visiting the emergency room of his primary care doctor. The patient had no history of raw food ingestion, fever, diarrhea, hematochezia, or exposure to organic solvents.

**FIGURE 1:**
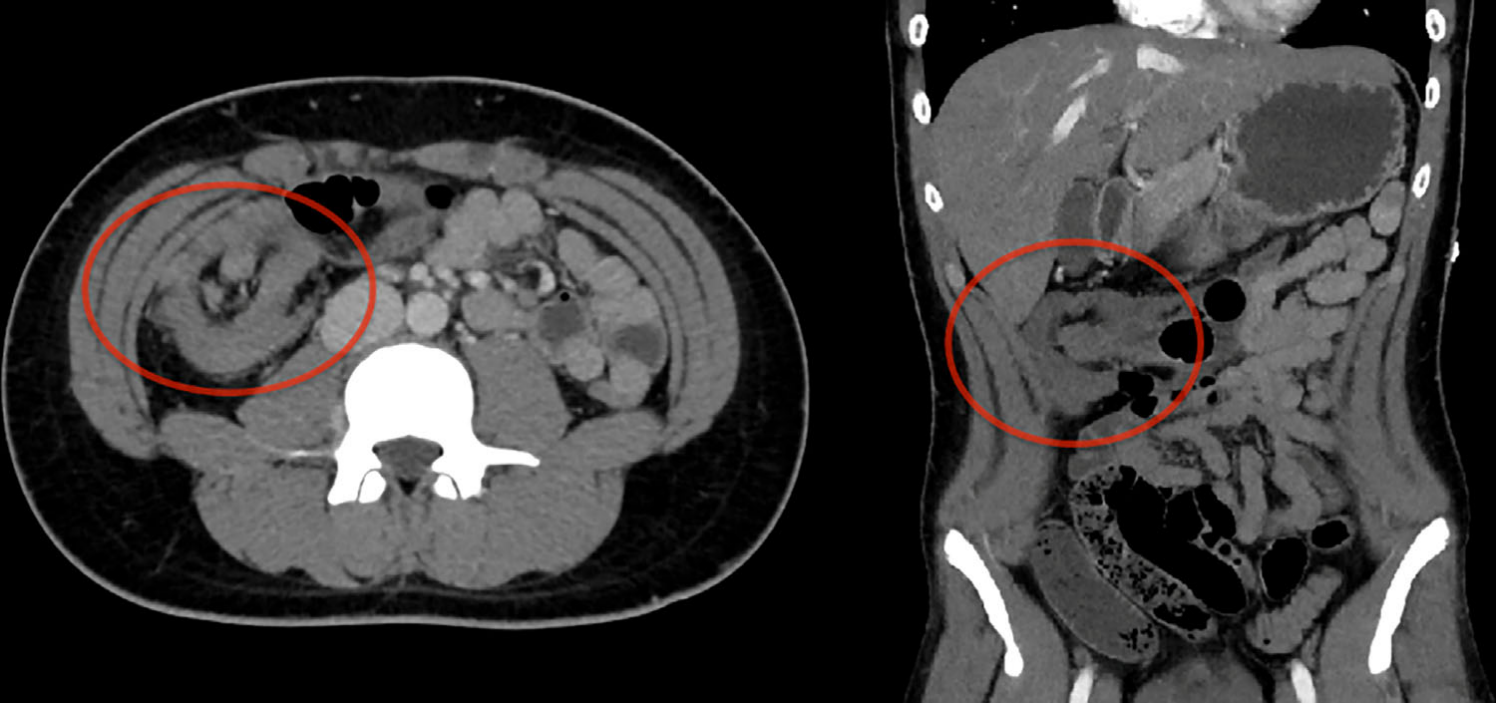
Computed tomography image on admission shows a target sign in the ileocecal region, indicating intussusception (red circle).

On admission, his vital signs were normal, and his abdomen was soft, non‐distended, and non‐peritonitic, with mild tenderness in the lower abdomen. Blood tests revealed a mildly elevated C‐reactive protein level of 2.5 mg/dl, but no other abnormal findings were noted. Computed tomography (CT) of the abdomen showed a target sign in the terminal ileum, suggesting intussusception (Figure 1).

**FIGURE 2:**
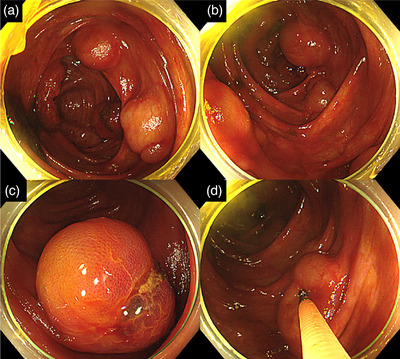
Colonoscopy: (a) Multiple smooth, elevated lesions on the mucosal surface in the cecum. (b) Multiple smooth, elevated lesions on the mucosal surface in the ascending colon. (c) Mucosal defects in the pneumatosis cystoides intestinalis. (d) Lesions are soft and show depression when pressed with biopsy forceps.

**FIGURE 3:**
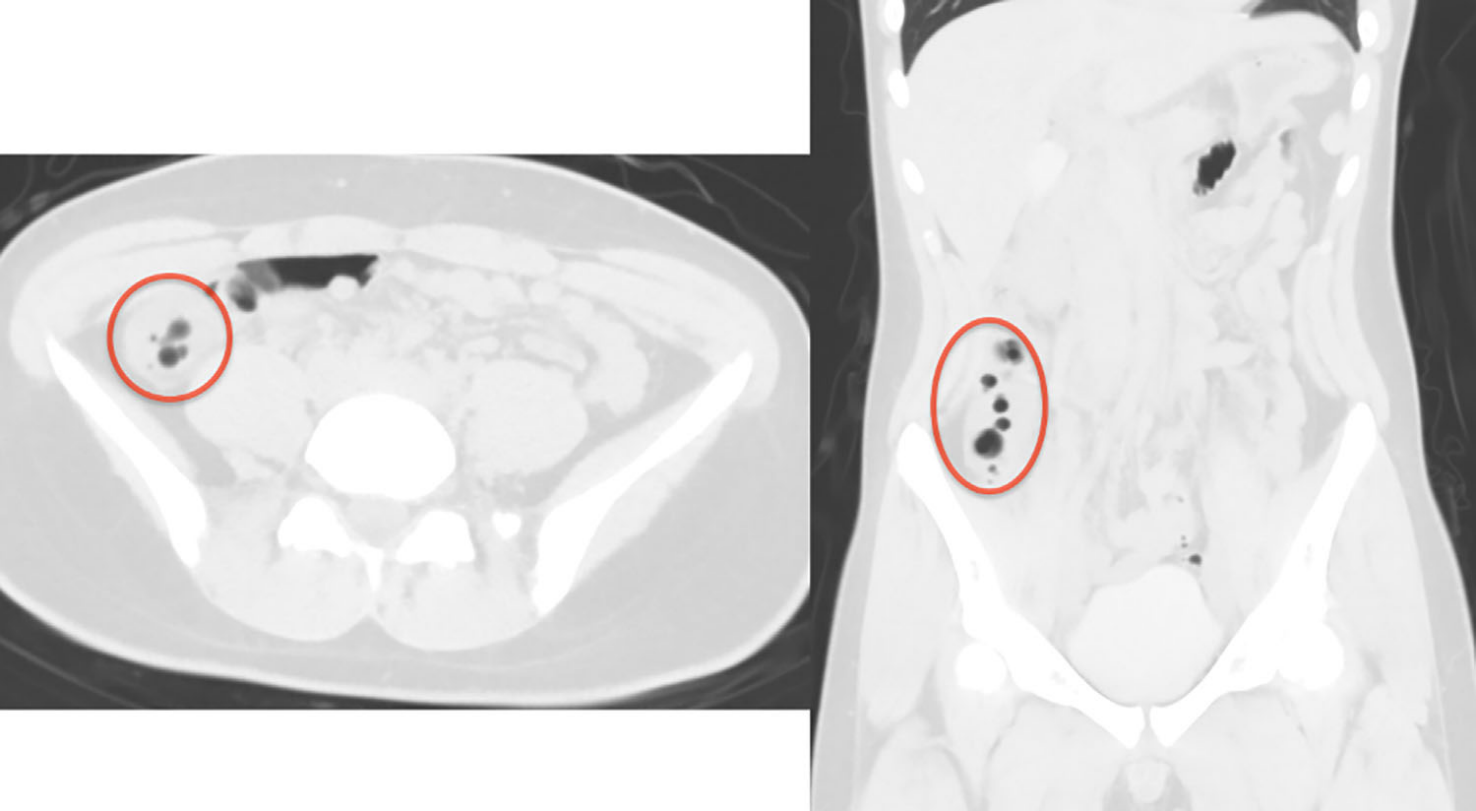
Computed tomography image (lung window) immediately after endoscopy showing findings of multiple spherical emphysematous lesions in the wall of the ascending colon (red circle).

Additionally, multiple spherical emphysematous lesions were found in the ascending colonic wall. CT showed no obvious gastrointestinal perforation or bleeding. A high‐pressure enema was administered immediately after admission, and a colonoscopy was performed 1 h later. By the time the scope reached the ascending colon during colonoscopy, the intussusception had resolved. The colonic mucosa on the anal side of the intussusception showed erythema without necrosis. However, multiple smooth, elevated, translucent lesions were observed in the ascending colon and cecum. These multiple elevated lesions depressed when pressed with biopsy forceps (Figure 2). The most common cause of intestinal calculus in adolescents is lymphoma. While endoscopic findings suggested lymphoma as a differential diagnosis, the diagnosis of PCI was made based on the CT images showing emphysematous lesions (Figure 3) and the above‐described multiple scattered masses on endoscopy. Barium enteroscopy was not performed. There were no findings suggestive of malignancy. Immediately after the colonoscopy, a CT scan confirmed intussusception and PCI.

The intussusception had resolved. However, the cystic emphysematous lesions scattered throughout the ascending colon, which is suggestive of PCI, clearly remained (Figure 3). After recovery, the patient was gradually fed and had no recurrent abdominal pain or hematochezia.

**FIGURE 4:**
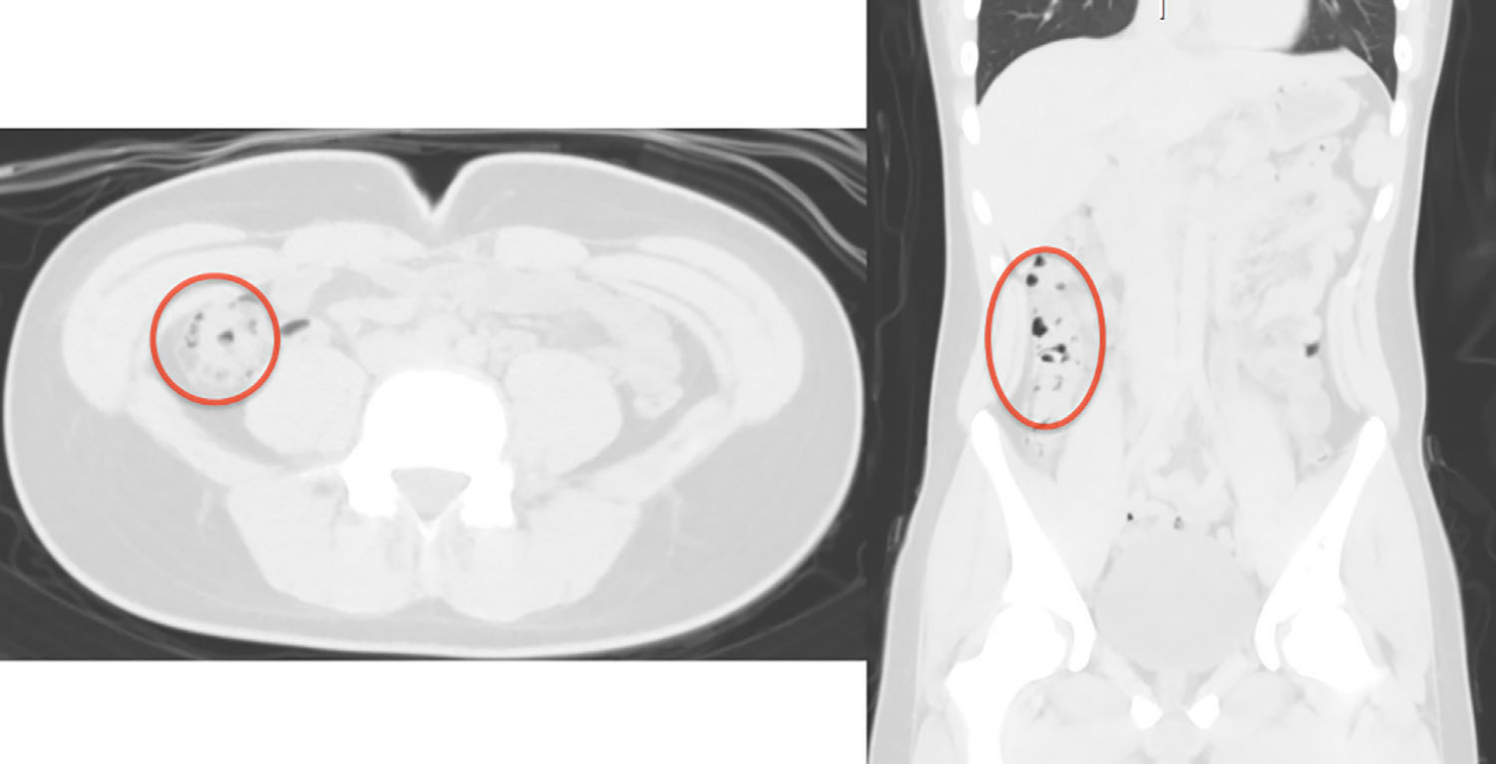
Computed tomography image on day 10 of reconstruction showing the emphysematous lesions had shrunk significantly (red circle).

As part of the treatment for PCI, oxygen was administered at 2 L/min during hospitalization. Although confirmation endoscopy could not be performed owing to the patient's refusal, follow‐up CT on day 10 after recovery showed that the PCI had decreased (Figure 4). The patient was discharged after remission was confirmed. After discharge, the patient was followed up in an outpatient clinic. Although no imaging evaluation was performed, there was no symptom recurrence after 1 year.

## DISCUSSION

We report intussusception associated with PCI in a male adolescent. Intussusception associated with PCI was treated with hyperbaric enema and low‐flow oxygen therapy.

PCI can be classified as idiopathic or secondary to an underlying disease. Koss et al. reported that idiopathic and secondary cases account for 15% and 85% of all cases, respectively.^5^

Secondary cases include collagen diseases, chronic obstructive pulmonary disease, systemic lupus erythematosus, inflammatory bowel disease, malignant tumors, and long‐term steroid, α‐glucosidase inhibitor (α‐GI), and chemotherapy use.^5^ In our patient, there were no underlying diseases. The reason why secondary PCI is common in Japan is the high rate of α‐GI use. In Japan, α‐GI, which is rarely administered in Western countries where fat accounts for a larger proportion of caloric intake than carbohydrates, is administered in approximately 30% of patients. According to Tsujimoto et al., 13 of 14 reported cases of α‐GI‐related PCI were from Japan.^6^ As idiopathic PCI and even intussusception associated with PCI are very infrequently reported, it is difficult to say that there is a statistical excess, but the few reports we found in PubMed were from Japan. Intussusception associated with PCI may be more commonly observed in Japan, where CT imaging is overwhelmingly more common, although endoscopic and CT imaging findings are very powerful clues.

Intussusception is generally a disease that occurs in infants with loose fixation of the mesocolon, and adult cases are rare. Idiopathic intussusception in infants is more common (75%), and only 25% of cases are due to a pathological lead point (PLP). In adults, PLPs, such as a tumor or Meckel's diverticulum, have been reported in 90% of cases.^7^ In this case, the PCI in combination with a mucosal defect found therein (Figure 2c) was considered the likely PLP for the patient's intussusception.

Oxygen inhalation therapy is a PCI treatment, and hyperbaric oxygen therapy is particularly effective.^8^ High‐concentration oxygen inhalation increases arterial oxygen concentration, which displaces the nitrogen in the emphysematous lesions. The replaced oxygen is absorbed by the tissues and capillaries, resulting in the resolution of emphysema. However, hyperbaric oxygen therapy is not widely available. As it was unavailable in our hospital, we administered oxygen at 2 L/min and observed for PCI resolution. Miyazato et al. reported a PCI case, in which the patient's condition was improved with low‐flow oxygen inhalation (at 2 L/min).^9^

Although there are very few reports of intussusception associated with PCI, several characteristics become clear when considering the reports to date. First, intussusception associated with PCI has mainly been reported in Japan. Based on 33 cases published in Japan between 1983 and 2020, Nomura et al reported that most patients with PCI alone had a mean age of onset of 60–80 years, without sex difference; moreover, idiopathic cases were more common in the left colon. In contrast, the mean age of onset of PCI‐associated bowel calculus was ≤18 years and the condition was more common in male individuals. Additionally, all cases had emphysema in the area including the right‐sided colon.^10^ Our case was also consistent with these findings, occurring in the right‐sided colon in a 16‐year‐old male. Analysis of the 33 cases showed that 11 patients underwent surgery (bowel resection) in the initial treatment, while 11 patients underwent surgery in the secondary treatment due to recurrence after conservative treatment, such as high‐pressure enema and endoscopic resection in the initial treatment (total 22 cases [66.7%]). Of the 13 patients who received only conservative treatment and no oxygen as first‐line treatment, 10 underwent surgery as second‐line treatment, while only one of the 10 patients who received hyperbaric oxygen or high‐concentration oxygen in addition to conservative treatment required surgery.

These results suggest that intussusception associated with PCI occurs at an average age of 18 years and that bowel resection is required in 67% of patients. Nevertheless, surgery is significantly more likely to be avoided in patients who receive conservative treatment plus oxygen.

Although hyperbaric oxygen therapy has been reported to be a useful treatment for PCI, the reason many patients do not receive adequate oxygen therapy and are forced to undergo surgery may be due to the extremely high threshold for hyperbaric oxygen therapy in Japan. Therefore, treatment with low‐flow oxygen therapy, as described in this case, is very useful. We propose initial treatment with low‐flow oxygen therapy for patients with PCI and mild symptoms, followed by high‐flow oxygen therapy when the patient becomes unresponsive. Hyperbaric enema and low‐flow oxygen therapy are available in most hospitals and could be the first‐line therapy for patients with PCI, but further case series and comparative studies are warranted.

In conclusion, we report a case of juvenile‐onset intussusception with advanced PCI, which was treated using a high‐pressure enema and low‐flow oxygen therapy. Intermittent abdominal pain without diarrhea or hematochezia in male adolescents should prompt the inclusion of intussusception due to PCI in the differential diagnoses, and after diagnosis, conservative therapy combined with oxygen therapy should be considered after confirming the absence of adverse findings, such as intestinal ischemia, perforation, or bleeding.

## CONFLICT OF INTEREST STATEMENT

None.

## ETHICS STATEMENT

This study was conducted in accordance with the Declaration of Helsinki and with approval by the relevant institutional review board. The patient provided informed consent for the publication of this report.

## Data Availability

No data was generated in the writing of this case report.
